# Scientific Medicine in Its Relation to Homœopathy

**Published:** 1891-12

**Authors:** 


Scientific Medicine in its Relation to Homœopathy. By Professor Theodor Bakody, M. D., of the University of Buda-Pesth. Translated from the German by Rudolph F. Bauer, M. D. Philadelphia: Boeriche & Tafel, 1891. Price, 50 cents.

This beautifully printed and bound little volume of 60 pages is very interesting. We have read it from beginning to end, and would advise our physicians to procure the book and judge for themselves.

It appears that Professor Bakody, reared in the allopathic school, began to investigate Homœopathy, and was appointed Professor of Homœo-therapeutics by the Hungarian government. The Buda-Pesth University is, as a matter of course, old school, and Homœopathy is taught as a kind of an "addition," to be accepted or rejected by the students, just as it is at the University of Greifswald.

We would not for one moment think of detracting from the honors and learning of Professor Bakody and his great work at the University, but cannot refrain from remarking that to judge by this book, he has not fully grasped the sublime truths of Homœopathy. But let us hope that he is earnestly seeking, and "he that seeketh shall find, and unto him that knocketh, it shall be opened." W. S.

